# Characterizing stay-green in barley across diverse environments: unveiling novel haplotypes

**DOI:** 10.1007/s00122-024-04612-1

**Published:** 2024-05-06

**Authors:** Stephanie M. Brunner, Eric Dinglasan, Silvina Baraibar, Samir Alahmad, Christina Katsikis, Sarah van der Meer, Jayfred Godoy, David Moody, Millicent Smith, Lee Hickey, Hannah Robinson

**Affiliations:** 1 Queensland Alliance for Agriculture and Food Innovation, The University of Queensland, St Lucia, QLD Australia; 2 InterGrain Pty Ltd, Perth, WA 6163 Australia; 3 School of Agriculture and Food Sustainability, The University of Queensland, Gatton, QLD Australia

## Abstract

**Key message:**

There is variation in stay-green within barley breeding germplasm, influenced by multiple haplotypes and environmental conditions. The positive genetic correlation between stay-green and yield across multiple environments highlights the potential as a future breeding target.

**Abstract:**

Barley is considered one of the most naturally resilient crops making it an excellent candidate to dissect the genetics of drought adaptive component traits. Stay-green, is thought to contribute to drought adaptation, in which the photosynthetic machinery is maintained for a longer period post-anthesis increasing the photosynthetic duration of the plant. In other cereal crops, including wheat, stay-green has been linked to increased yield under water-limited conditions. Utilizing a panel of diverse barley breeding lines from a commercial breeding program we aimed to characterize stay-green in four environments across two years. Spatiotemporal modeling was used to accurately model senescence patterns from flowering to maturity characterizing the variation for stay-green in barley for the first time. Environmental effects were identified, and multi-environment trait analysis was performed for stay-green characteristics during grain filling. A consistently positive genetic correlation was found between yield and stay-green. Twenty-two chromosomal regions with large effect haplotypes were identified across and within environment types, with ten being identified in multiple environments. In silico stacking of multiple desirable haplotypes showed an opportunity to improve the stay-green phenotype through targeted breeding. This study is the first of its kind to model barley stay-green in a large breeding panel and has detected novel, stable and environment specific haplotypes. This provides a platform for breeders to develop Australian barley with custom senescence profiles for improved drought adaptation.

**Supplementary Information:**

The online version contains supplementary material available at 10.1007/s00122-024-04612-1.

## Introduction

Barley (*Hordeum vulgare*) is the fourth most important cereal crop based on area and production globally and possesses drought adaptation mechanisms, which makes it an excellent candidate for further improving the resilience of farming systems (Badea and Wijekoon 2021). With climate change causing increased temperatures and decreased winter rainfall there is an urgent imperative to breed more environmentally adapted crops. Drought tolerance is complex and composed of many component traits with both optimization of above and below ground physiological pathways driving yield (Borrell et al. 2014).

Stay-green is a key trait involved in drought adaptation of crops. Stay-green specifically refers to the onset of senescence post-anthesis. During senescence, the photosynthetic apparatus of the leaf are broken down into peptides that can be transported through the phloem to the developing grain (Feller and Fischer 2011). This regulated process of cell breakdown is the cause of the observed color change of crops and other plants from green to brown. Thus, genotypes that display stay-green under drought conditions are referred to as ‘stay-green’ types (Borrell et al. 2000). The stay-green phenotype is caused by the intersection of multiple traits. In sorghum, key chromosomal regions for stay-green aid in reducing tillering by increasing size of lower leaves and constraining upper leaf area reducing early season water demand while reducing root angle to produce more roots at depth and increase access to stored soil moisture (Borrell et al. 2014). While stay-green has proven to be a valuable trait for drought adaptation in sorghum (Borrell et al. 2000), maize (Yang et al. 2017), and wheat (Christopher et al. 2016), the trait is yet to be fully explored in barley.

Characterization of stay-green relies on the use of indirect measurements of leaf chlorophyll concentration, which can be achieved through a variety of approaches. Vegetative indices, such as normalized difference vegetation index (NDVI) and normalized difference red edge index (NDRE), can be measured by Greenseeker® or multispectral imagery and are presently the best non-destructive means of ascertaining chlorophyll concentration (Liedtke et al. 2020). New advances in the use of unmanned aerial vehicles (UAVs), multispectral cameras, and high throughput phenotyping have provided an opportunity to better understand the nature and variation of post-anthesis senescence in barley (Liu et al. 2022; Panday et al. 2020). This technology allows for precise and accurate capture vegetative indices, decreased probability of operator error, and significantly decreased operating time allowing for more frequent measurements to be taken. While vegetative indices provide excellent proxy measures for stay-green, it is important to maintain an awareness that a persistence of green canopy color can also be attributed to cosmetic differences rather than maintenance of photosynthetic machinery and therefore a prolonged grain filling period (Balazadeh 2014; Borrell et al. 2014; Liang et al. 2017). The implementation of UAVs will allow for more robust stay-green phenotyping which is key to dissecting stay-green and its role in barley.

The process of senescence overlaps with the grain filling phase of development as nutrients are remobilized from the leaf to the developing grain. Consequently, stay-green has been linked to increased yield in sorghum, maize, wheat, and rice as it increases the photoassimilate available for grain fill (Borrell et al. 2014; Liang et al. 2017; Shin et al. 2020; Zhang et al. 2021). Stay-green has been extensively researched in cereals for its potential to increase yield under water-limited conditions (Kamal et al. 2019). Lope and Reynolds (2012) found that in both heat and drought stress environments stay-green traits can account for 26% and 36% of wheat yield variation, respectively. However, studies in wheat conducted in well-watered conditions have observed a negative relationship between yield and maintenance of green leaf area suggesting that tradeoffs are likely to be associated with stay-green in some environments (Derkx et al. 2012; Kipp et al. 2014).

While this trait is well-studied in wheat, minimal research has been conducted in barley. Stay-green in barley has been linked to an increased grain filling period. However, there is little evidence of a positive or negative correlation with yield due to a lack of research (Shirdelmoghanloo et al. 2022; Williams et al. 2022). Emebiri (2013) found that retention of green leaf area can result in an increase in grain plumpness and yield. However, this study examined two bi-parental populations in two well-watered environments, which limits the ability to draw broad conclusions regarding the impact of stay-green on yield in a wider array of environmental conditions and across more diverse germplasm. Thus, given the importance of source–sink relationship during senescence and the critical grain filling period, understanding the value of this trait to support yield can help inform decision making in breeding programs for step changes to this key trait.

Not only is the variation and value of stay-green unknown in barley but there is limited research on the genetic controls of the trait. Understanding the genetic controls of stay-green will enable more efficient breeding through a range of genomics-assisted breeding strategies. Emebiri (2013) analyzed two bi-parental barley populations Vlamingh/Buloke and VB9524/ND11231*12 for stay-green QTLs. Phenotyping was conducted using a handheld optical sensor twice during the growth cycle, both pre- and post-maturity, to calculate a change in ratio of visual to near-infrared light reflection. Across environments, only one QTL was consistently detected on chromosome 5H (bin06), which accounted for 5–15.4% of total variation (Emebiri 2013). As a result, the author hypothesized that the trait was governed by a small number of loci. In contrast to Emebiri’s findings, Gous et al. (2016) identified 10 QTL regions associated with stay-green in the ND24260-1/Flagship double haploid population. A key limitation of this study is that phenotyping was conducted under controlled environment conditions, making it difficult to capture the dynamics of water supply–demand that are critical for trait expression under field conditions. The use of bi-parental populations in both studies increases the power to detect rare QTL, but overall limits the genetic diversity assessed and the ability to detect QTL influencing the trait that exists outside of the two parents, limiting the translation to commercial breeding. Additionally, the modeling approaches restricted the traits available for analysis to single time point or rate comparisons. To assist plant breeders, characterization of stay-green’s genetic control is needed across a diverse panel of lines that are representative of commercial breeding. Furthermore,  a smoothed modeling approach is well-suited for capturing this dynamic process over time and enables extraction of different features including area under the curve and derivatives (Perez-Valencia et al. 2022). A further consideration for QTL mapping or genome-wide association studies (GWAS), is the utility of single trait-associated markers for breeding applications. Single markers can lose linkage with the desired trait through recombination, however, the identification of haplotypes containing multiple markers in high linkage disequilibrium does not have the same issue. One haplotype-based association approach utilizes local genomic estimated breeding values (LGEBVs) to determine the value of different haplotypes on a trait of interest (Voss-Fels et al. 2019). The identification of haplotypes contributing to stay-green provides more trackable and achievable targets for plant breeders who wish to select for the trait (Voss-Fels et al. 2019).

Using modern phenomics and genomics technologies, this study aims to build on the foundational knowledge of the genetic control of stay-green in barley. Specifically, the study aims to; (1) characterize the phenotypic variation in stay-green across a large barley breeding panel, (2) explore the importance of environment on stay-green specifically during the grain filling period and the relationship with yield, and (3) identify key haplotypes associated with stay-green across a range of growing environments.

## Materials and methods

### Plant materials

This study examined a panel of 397 two-rowed spring barley genotypes that are representative of historic and modern breeding germplasm from InterGrain Pty Ltd, an Australian commercial cereal breeding company. The panel was genotyped with the Infinium™ wheat barley 40K v1.0 BeadChip, returning 12,561 high quality single nucleotide polymorphism (SNP) markers (Keeble-Gagnere et al. 2021).

### Field trials

The panel comprising 397 genotypes was evaluated for yield and days to anthesis in six field trials conducted across Western Australia (WA) and Queensland (QLD) during the 2022 and 2021 growing seasons (Table 1). The check cultivar Maximus CL was scored for growth stage prior to each UAV flight to provide an indication of development progression. Each trial was sown as two by six-meter plots cut back to 10 m^2^ with partial replication in a randomized block design. All trials experienced rain fed conditions and insects and weeds were controlled as required. Weather data was collected by weather stations set up adjacent to the trail location for Corrigin, York, and Gatton. Weather data for Dandaragan was taken from the SILO database station number ‘9014—West Dandaragan’ (Jeffrey et al. 2001).

**Table 1:** Trial information with coordinates, sowing data and number of post-flowering UAV flights

Trial Code	Sowing date	Location	Trial coordinates	Post-flowering UAV flights
21CGN	23/05/2021	Corrigin, WA	− 32.3682, 117.94028	3
21DND	12/05/2021	Dandaragan, WA	− 30.7519, 115.66832	3
22CGN	26/05/2022	Corrigin, WA	− 32.38002, 117.94482	3
22GAT	16/06/2022	Gatton, QLD	− 27.5496, 152.3579	7
22DND	6/05/2022	Dandaragan, WA	− 30.65153, 115.7843	4
22YRK	19/05/2022	York, WA	− 32.02549, 116.86265	3

### UAV phenotyping

All field trials were subjected to remote sensing using a Matrice300 unmanned aerial vehicle (UAV) mounted with a multispectral MicaSense Altum™ camera, which captured images across five wavelength bands, red, green, blue, red edge, and near-infrared. Images were captured at 20 m above each trial field for approximately 30 min within two hours of solar noon. An overlap of 80% with ground control points for accurate determination of plot location and calibration was conducted to maintain flight course by scanning a quick response code. Notably, the number of flights per location was dependent on weather, manpower, and time. Between 3 and 7 flights were conducted per trial location from anthesis through to crop maturity. Flights were deemed to be *post-flowering* if the Zadoks of commercial cultivar Maximus CL was greater than 49, indicating awn peep, at the time of the flight (Zadoks et al. 1974).

Agisoft Metashape (Agisoft LLC, St. Petersburg, Russia) software was utilized to stitch images, perform camera calibration, and check for accuracy resulting in an orthomosaic for each flight. A shape file corresponding to the experimental design was created using ArcGIS software. The orthomosaic and shape files were combined in Xtractori (Das et al. 2022) for extraction of vegetative indices including, NDVI and NDRE.

At maturity, yield was determined using a machine plot harvester which calculated yield as weight per unit area and later converted to tones per hectare.

### Longitudinal modeling and statistical analysis

NDRE was selected as the vegetative index for further analysis due to its strength in measuring chlorophyll concentration for a closed canopy (Anderegg et al. 2020). A pseudo-two stage longitudinal modeling approach was selected to correct for spatial and temporal variation of the raw NDRE data and model canopy senescence over time. This modeling was conducted using the statgenHTP package (Perez-Valencia et al. 2022) in R software (R Development Core Team 2023). Both the mixed linear model and two-dimensional penalized spline (P-spline) models (SpATs) approaches were compared for their ability to account for the spatiotemporal variability across all time points for each environment. The linear mixed model was fitted using ASReml- R (Butler et al. 2009) with an autoregressive residual variance structure in the order of one, with model ‘best fit’ determined by the Akaike information criteria (AIC; Akaike 1974). The linear mixed model equation follows:

$$y = X\tau + Zu + e$$

where *X* and *Z* are design matrices associated with fixed (*τ*), and random (*u*) effects and *e* is the residual errors.

The SpATs model was fitted for each time point with the following equation:

$$y\left( t \right) = 1_{M} \beta_{0t} + X\beta_{t} + Zc_{t} + Z_{g} c_{gt} + f_{t} \left( {u,v} \right) + \varepsilon_{t} , c_{t} {{\sim }}N\left( {0,G_{t} } \right), c_{gt} {{\sim }} N\left( {0,G_{gt} } \right), \varepsilon_{t } {{\sim }} N\left( {0, \sigma^{2} I_{m} } \right)$$

where ${f}_{t}\left(u,v\right)$ is the smooth function of the spatial trend, ${1}_{M}{\beta }_{0t}$ is a vector of NDRE values and accompanying vector of 1s, ${X\beta }_{t}$ represents the factors present in the model, *G* is a matrix of smoothing components and variance parameters, *c* represents the random effects associated by time (*t*) and genotype (*g*) with associated *Z* design matrices and $\varepsilon_{t }$ is the error variance.

Spatial variation in each environment was modeled by both approaches. To determine the most appropriate model for the spatial variation in each environment the broad-sense heritability was calculated for all time points in both models. The model with the highest broad-sense heritability for the trait across the greatest number of time points was selected due to its ability to account for the most spatial variance. To account for flowering time, genotypes in each environment were placed into even groups based on similar flowering times. This flowering grouping was then added to the model as a fixed effect.

A three-level nested hierarchical growth model was fitted to the corrected NDRE values for all genotypes (Perez-Valencia et al. 2022) as per:

$$y_{pgi} \left( t \right) = f_{p} \left( t \right) + f_{pg} \left( t \right) + f_{pgi} \left( t \right) + \varepsilon_{pgi} \left( t \right), N{{\sim }}\left( {o,\sigma^{2} \omega_{pgi} \left( t \right)} \right)$$

where *f*_*p*_ is the change in NDRE overtime for spatially corrected phenotype for the *p*-th population, *f*_*pg*_ is the deviation from *f*_*p*_ due to genotype, *f*_*pgi*_ is the deviation from *f*_*g*_ due for individuals and $\varepsilon_{pgi}$ is the associated error.

The degree of B-spline basis was set at three with a penalty order of two with seven knots. This enabled the determination of P-splined growth curves of NDRE modeled for senescence from anthesis to harvest over thermal time for the 22GAT environment and during the key grain filling period for all environments. From these curves multiple time-NDRE traits were extracted including the rate, area under the curve and area under the curve of the first derivative.

This approach was selected for its ability to incorporate multiple time points of UAV data into a single, smoothed, and dynamic model of the observed values. In addition, the use of spatiotemporal longitudinal modeling accounts for environmental effects in the temporal dimension as well as the spatial which is a limitation of parametric models (Perez-Valencia et al. 2022). The derivatives of P-splined growth curves are easily calculated providing greater ability to dissect the trait and distinguish different types of stay-green.

Yield data underwent spatial analysis through application of a linear mixed model utilizing ASReml-R (Butler et al. 2009). Column, row, and replicate were fitted as random effects along with an autoregressive residual variance structure in the order of one. Model fit terms were deemed significant by REML log-likelihood ratio and the AIC statistic. A generalized measure of broad-sense heritability was determined for each environment as per:

$$h_{g}^{2} = 1 - A_{tt} {/}\left( {2\gamma_{{\text{v}}} } \right)$$

where *A*_*tt*_ is the average prediction error variance and $\gamma_{{\text{v}}}$ is the genetic variance.

Best linear unbiased estimators (BLUEs) were estimated from the spatial model by fitting genotype as a fixed effect in the linear mixed model with a variance structure of autoregressive one.

### Multi-environment trial analyses

The genotype by environment (G × E) interactions were explored for total area under the curve for NDRE during grain filling (scsAUC) using a two stage multi-environment trait analysis (MET) using the BLUEs from the longitudinal modeling curves. As spatial correction had already been completed, no trial-specific effects were included in the analysis. A diagonal model and correlation models with assumptions of homogeneous and heterogeneous variation were fitted to the data to identify the best model for the G × E interactions. After determining that the genetic variances and co-variances were not equal between sites a factor analytic (FA) variance structure was fitted for the G × E interactions (Smith et al. 2001). The number of factors applied within this structure was increased until the optimum model was identified utilizing REML log-likelihood, the AIC and the total observed genetic variance accounted for by the model. The genotypic effect and its associated variance was calculated using the following equation:

$$u_{g} = \left( {\lambda_{1} \otimes I_{m} } \right)f_{1} + \left( {\lambda_{2} \otimes I_{m} } \right)f_{2} + \left( {\lambda_{3} \otimes I_{m} } \right)f_{3} + \delta$$

where the coefficient *λ*_*i*_ are the known trial site loadings,  *I*_*m*_ represents the genetic variance matrix and *δ* is the vector of residuals (Smith et al. 2001).

 Following the final linear model fit, interaction classes (iClasses) were identified, and determined by the polarity of the estimated environmental loadings for each factor from the final FA3 model.. Within each iClass crossover G × E is minimal as genotype rankings remain similar, BLUEs were then calculated from the MET variance components for each iClass (Smith et al. 2015).

To calculate genetic correlations, BLUEs for each environment from the yield and scsAUC analysis were combined in a multi-trait two-stage MET approach. A genomic relationship matrix was calculated using R package ‘AGHmatrix’ (Amadeu et al. 2023) and the inverse fitted as a random effect in the model. The best model was identified using the REML log-likelihood and AIC values and correlations between scsAUC and yield determined for each environment were calculated based on the model. Phenotypic correlations between yield and scsAUC were determined using linear regression.

### Haploblock discovery, and haplotype stacking analyses

Curation of the 12,561 SNP markers was performed to remove markers with a minor allele frequency of less than or equal to 0.05 and those with greater than 10% heterozygosity, resulting in a curated marker dataset comprising 6743 SNP markers. Principal component analysis was conducted utilizing a genetic distance matrix calculated with Rogers’ distance (Rogers 1972), to identify clusters of individuals from similar genetic backgrounds, utilizing R package ‘Selection Tools’ (Selection Tools Developers 2023). Examination of the dendrogram of relatedness between the genotypes was used to determine appropriate number of clusters present in the panel.

‘Selection Tools’ was also utilized to develop linkage disequilibrium (LD) blocks based on patterns of recombination present in the genotype data (Selection Tools Developers 2023). A total of 2857 LD blocks were classified by applying an LD threshold of 0.7 and a marker tolerance of 2, for each block the range of SNP combinations present in the population were called haplotypes. Marker tolerance is the number of SNPs below the set LD threshold that can be included in a block.

Detection of haplotypes was conducted for scsAUC utilizing the BLUEs from the MET analysis and individually for each iClass. Additionally, haplotype analysis was completed on the BLUEs for area under the rate curve (rAUC) for the entire 22GAT curve. For each haplotype, the local genomic estimated breeding value (i.e., localGEBV) was calculated (Voss-Fels et al. 2019) using a ridge-regression best linear unbiased prediction model (RRBLUP) to determine the effect of every SNP simultaneously (Meuwissen et al. 2001). As per the equation:

$$y = WGu + \varepsilon$$

where *u* is a vector of marker effects, *G* is the genotype matrix, *W* is a matrix relating genotypes to observations (*y*) and $\varepsilon$ is the error.

These effects were then summed based on the SNPs present in each haplotype to quantify the effect of each haplotype on AUC. The variance of effects of each block was determined and a scaled min–max variance threshold of 0.3 was used to determine block significance. Haplotypes were deemed beneficial if their effect was positive for scsAUC and negative for rAUC.

Haplotype ‘stacking’ was performed in silico to investigate the additive effects of multiple advantageous haplotypes to improve stay-green. This was carried out by determining the number of beneficial haplotypes present in each genotype for the subset of significant blocks. The average trait value for individuals grouped by number of blocks was then used to explore the effect of multiple haplotypes on the trait.

## Results

### High degree of genetic diversity in breeding panel

**Fig. 1:**
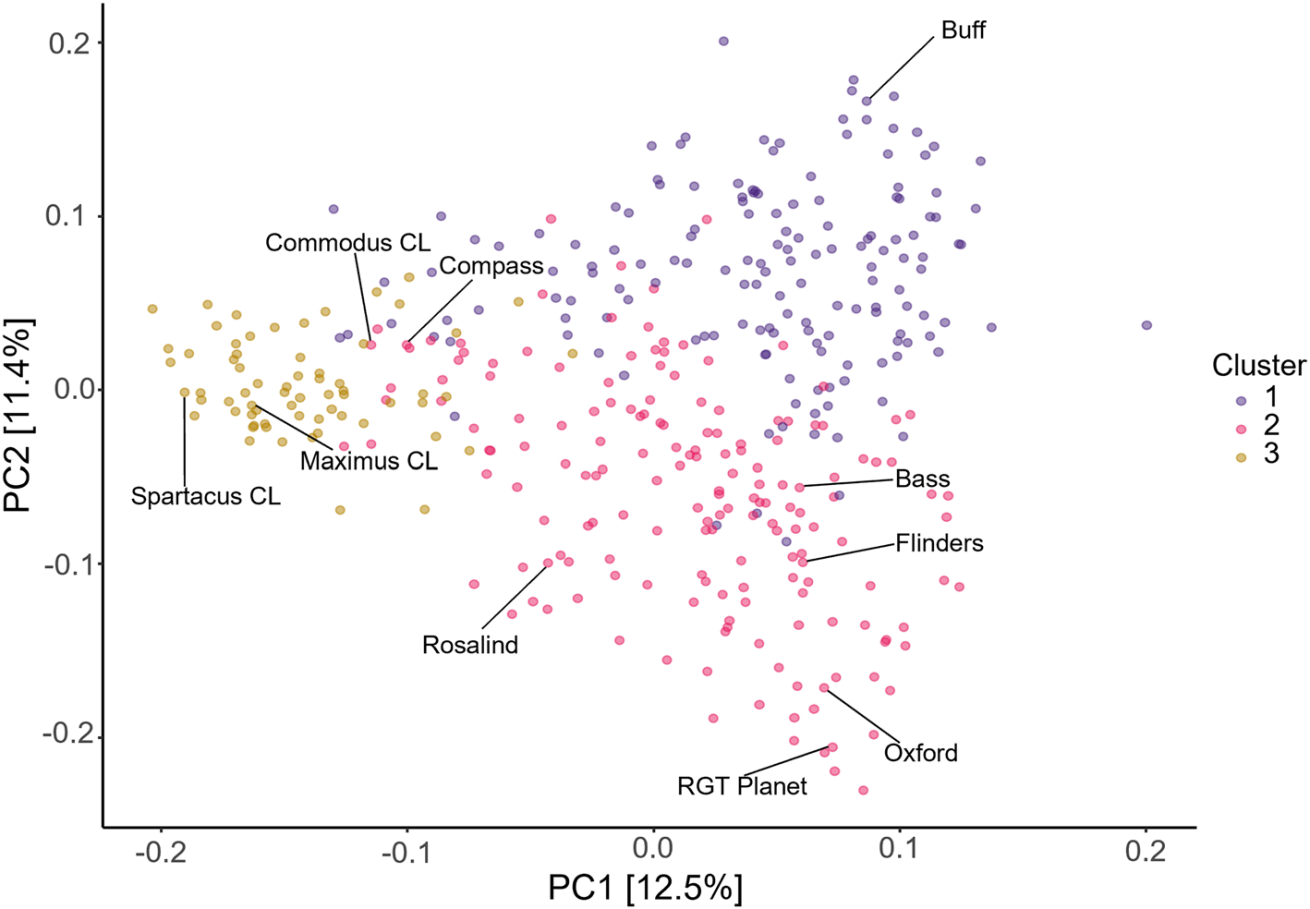
Population structure and genetic diversity of the barley breeding panel (*n* = 397). Principal component analysis was performed based on Roger’s distance and calculated using 6734 SNPs. The three distinct clusters are displayed in different colors. A subset of barley cultivars are labelled

The germplasm utilized in this study is genetically diverse and can be divided into three distinct clusters (Fig. 1). Principal components one and two account for 12.5% and 11.4% of genotypic variation present in the panel. The clusters align with historic Australian varieties (Cluster 1), European varieties (Cluster 2), and modern Australian varieties (Cluster 3).

### Large variation in senescence profile observed in barley breeding panel

**Fig. 2:**
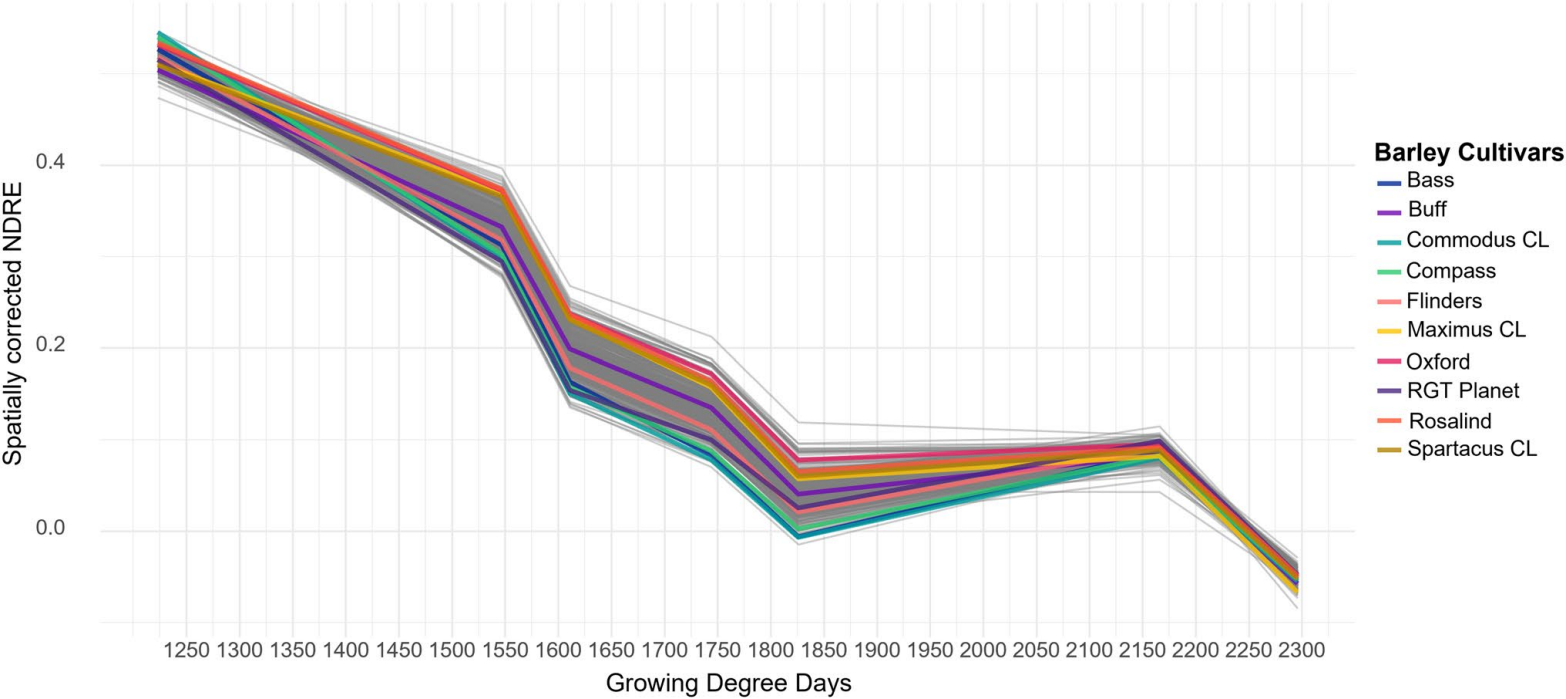
Senescence profiles of 397 barley varieties post-flowering to full maturity at Gatton in 2022. P-spline approach has been applied to data proving NDRE value for each day. A subset of commercial barley cultivars highlighted using different colors, as per the key

Variation in senescence profile was observed across barley genotypes in the 22GAT environment (Fig. 2). Variation was greatest during the central phase of 1600–1850 growing degree days, with a difference of 0.14 between the maximum and minimum NDRE values. This variation was also observed within elite barley cultivars with some varieties senescing quickly such as Commodus CL and Compass while others senesced more slowly such as Oxford and Rosalind.

**Fig. 3:**
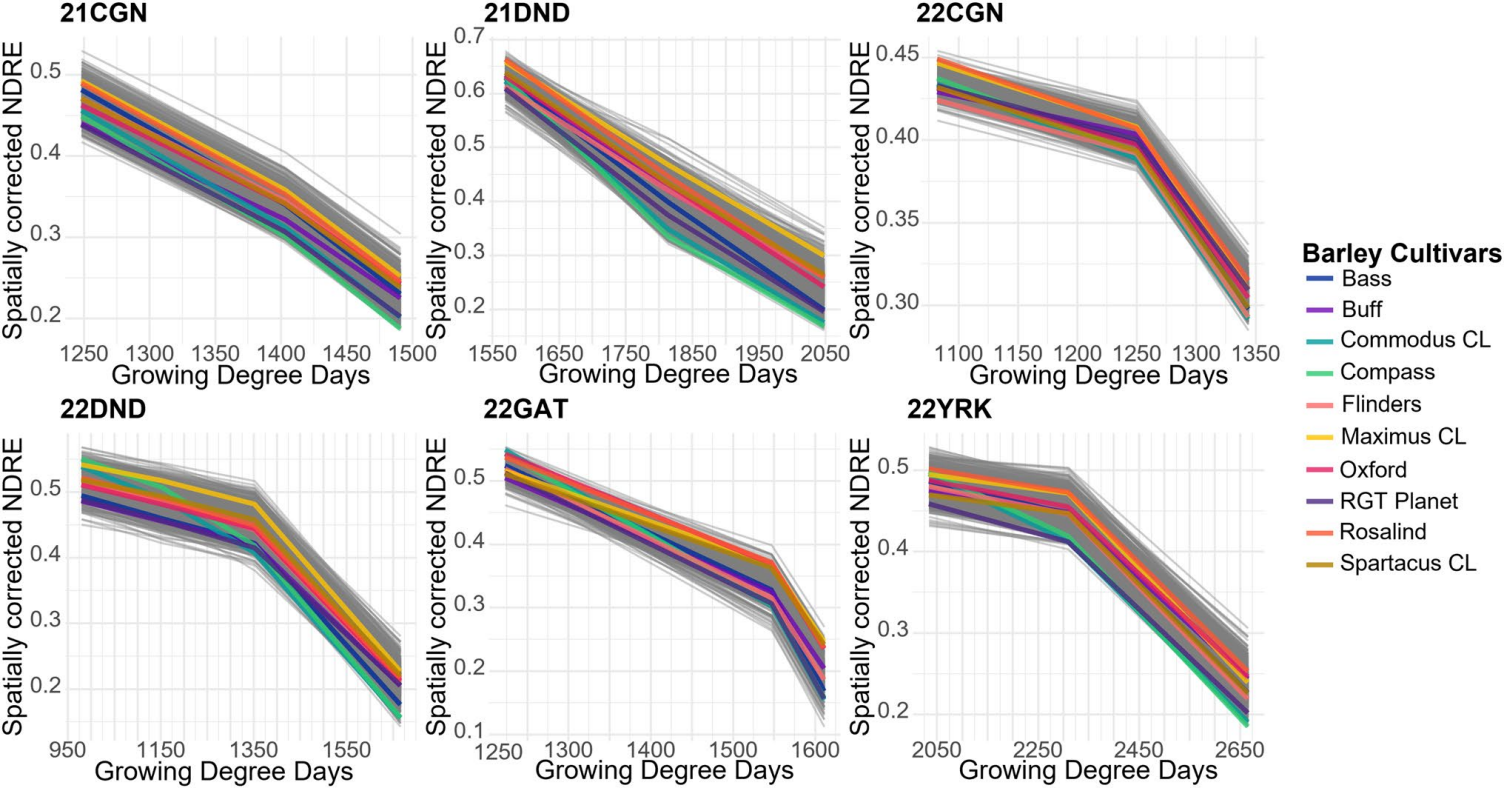
Senescence profiles of 397 barley varieties during grain filling across six locations. One location in Queensland (22GAT) and five in Western Australia. P-spline approach has been applied to data proving NDRE value for each day. A subset of commercial barley cultivars highlighted using different colors, as per the key

Similar variation was observed in the grain filling period across all six environments, however, varieties behaved differently under varying environmental conditions (Fig. 3). A range of rainfall and temperature profiles were seen across environments, however, rainfall across the sites was likely sufficient to circumvent severe drought conditions (Supplementary Fig. S1). Additionally, growth stage of Maximus CL did not align with the same thermal time across each environment.

### Variation due to genotype by environment interactions

**Fig. 4:**
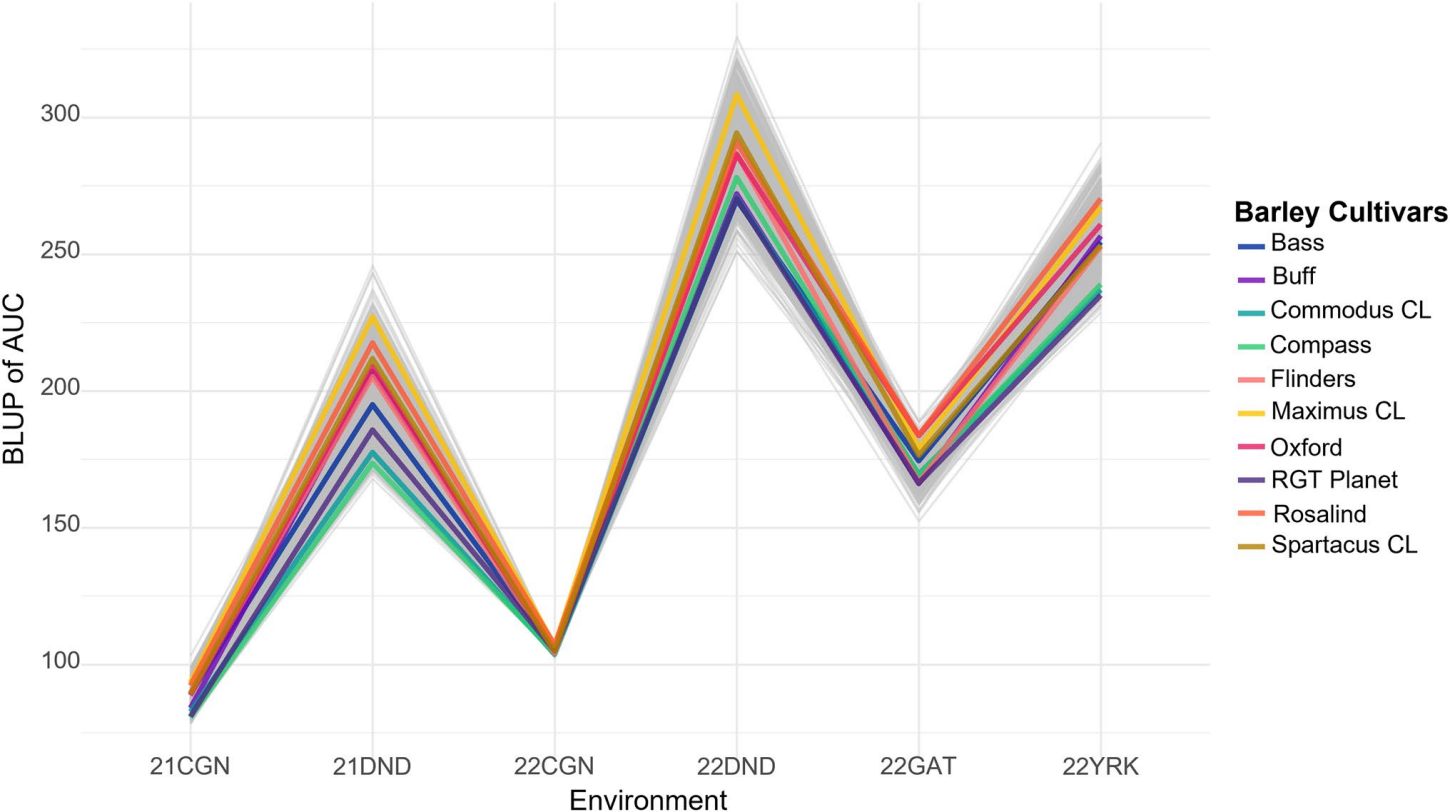
Genotype by environment interactions for senescence (area under the curve; AUC). Best linear unbiased predictors (BLUPs) calculated for the barley breeding panel across 6 environments. BLUPs were calculated using factor analytical modeling (FA3). Barley cultivars highlighted in different colors, as per the key

Environment contributes to variation in scsAUC with different environments showing different ranges in trait values. MET analysis identified a large amount of scale G × E interactions with some crossover G × E also identified (Fig. 4).

Factor analytic models were fitted for FA1 to FA5, to identify the model accounting for the most genetic variance (Smith et al. 2015). The FA3 model accounted for the greatest amount of genetic variance (61%) and was selected (Table 2).

**Table 2:** Goodness of fit for multi-environment trait analysis models for area under senescence curve

Model	AIC	REML Log-likelihood	% Variance accounted
DIAG	28,916.22	− 14,451.11	NA
CorV	32,950	− 16,472.83	NA
CorH	28,082	− 14,034.20	NA
FA1	28,003	− 13,989.53	47.58
FA2	28,006	− 13,985.78	59.01
**FA3**	**28,006**	**− 13,983.04**	**61.23**
FA4	28,014	− 13,983.04	56.65
FA5	28,012	− 13,983.04	57.55

Bold represents selected model

*DIAG* Diagonal model, *CorV* Correlation model with homogenous variation, *CorH* Correlation model with heterogeneous variation, *FA* Factor analytic models with one to five factors

The variance accounted for in each environment by each factor was not uniform with a total of 100% variance accounted for in 22DND and only 25.7% in 22CGN. Given the low broad-sense heritability of the 22CGN environment removal of this environment from the MET was trialed to determine if a better model could be identified. The removal of the environment did not improve the total variance accounted for by the FA model, and therefore the environment was kept for further analysis. The polarity of these factor loadings was utilized to identify three iClasses (Table 3).

**Table 3:** Parameters for each factor of the Factor analytic 3 (FA3) model

Environment	Factor 1		Factor 2		Factor 3		Total variance	H^2^	iClass
	Var	Loading	Var	Loading	Var	Loading			
21CGN	46.30	2.72	19.10	− 1.74	32.77	2.28	98.7	0.94	PNP
21DND	40.59	7.99	5.38	− 2.91	1.21	− 1.38	47.18	0.99	PNN
22CGN	10.90	0.37	10.91	− 0.37	3.90	− 0.22	25.72	0.56	PNN
22DND	93.05	12.29	6.93	3.35	0.02	0.18	100	0.99	PPP
22GAT	29.51	3.53	3.85	− 1.28	0.14	− 0.25	33.51	0.97	PNN
22YRK	27.29	5.49	2.23	− 1.57	0.38	0.65	29.91	0.99	PNP

Variance accounted for in each environment by each factor in FA3 model (Var), factor loading (Loading), broad-sense heritability (H^2^), and iClass

### Relationship between yield and stay-green varies depending on environment

The FA1 model was found to best model of environmental effects for determination of genetic correlation between scsAUC and yield based on REML log-likelihood and AIC (Table 4). Additionally, this model accounted for over 98% of genetic variance in the model. Yield was found to be positively correlated with scsAUC in all six environments. The genetic correlation ranged from 0.33 in 22CGN to 0.79 in 22DND (Table 5). The phenotypic correlations were also positive in all environments and ranged from 0.15 to 0.49.

**Table 4:** Goodness of fit for genetic correlation models

Model	REML Log-likelihood	AIC
Diag	− 15,240.53	30,505
Corh	− 14,982.24	29,655
Corv	− 28,995.07	57,994
**FA1**	− **14,746.72**	**29,553**

Bold represents selected model

*DIAG* Diagonal model, *CorV* Correlation model with homogenous variation, *CorH* Correlation model with heterogeneous variation, *FA*1 Factor analytic model 1

**Table 5:** Genetic and phenotypic correlations for yield and scsAUC in each environment

Environment	Genetic correlation	Phenotypic correlation
21CGN	0.71	0.29
21DND	0.68	0.30
22CGN	0.33	0.25
22DND	0.79	0.49
22GAT	0.57	0.15
22YRK	0.59	0.2

### Haplotypes associated with stay-green within and across environmental clusters

Novel haplotypes for scsAUC were detected within and across iClasses (Supplementary Fig. S2). Additionally, haplotypes were identified for rAUC from 22GAT and area under the senescence curve for the complete 22GAT senescence profile (GAT_scsAUC) (Supplementary Fig. S3). Each detection was conducted on a trait Senescence (scsAUC) or Rate (rAUC) within the environment (MET, PNN, PPP PNP, GAT).

Six chromosomal regions with high variation for haplotype effect were identified in the overall MET, ten in iClass PNN, eight in iClass PPP and five in iClass PNP. Further, six regions with high variation were identified for rAUC Gatton and two for GAT_scsAUC. Ten blocks with beneficial haplotypes were identified across multiple environments and traits (Table 6).

**Table 6:** Significant linkage disequilibrium (LD) blocks for stay-green traits. Blocks identified in multiple environment trait pairings

Chromosome	Position (bp)*	Block	Environment_trait
1	354,670,317	b000050	MET_scsAUC; PNP_scsAUC; PPP_scsAUC
2	5,032,278,825	b000453	GAT_rAUC; PPP_scsAUC
3	384,110,938	b000884	PNN_scsAUC; PPP_scsAUC
3	739,434,515	b000857	MET_scsAUC; PNP_scsAUC
3	4,530,142,495	b000894	MET_scsAUC; PNN_scsAUC; PNP_scsAUC
4	445,006,234	b001274	GAT_rAUC; GAT_scsAUC; PPP_scsAUC
5	378,715,777	b001605	GAT_rAUC; MET_scsAUC
5	2,128,254,105	b001588	GAT_scsAUC; PPP_scsAUC
6	483,484,376	b002278	MET_scsAUC; PNN_scsAUC; PNP_scsAUC; PPP_scsAUC
7	2,392,917,465	b002631	MET_scsAUC; PNP_scsAUC

*Position in base pairs as center of block

**Fig. 5:**
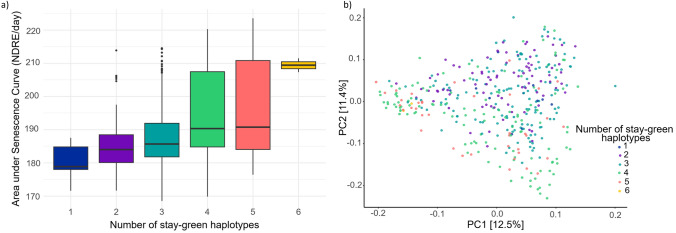
Haplotypes for area under the senescence curve (scsAUC) identified from best linear unbiased estimators across six environments. **a** AUC for individuals grouped by number of stay-green haplotypes, **b** diversity of genotypes in the barley breeding panel with number of stay-green haplotypes highlighted as per key. Principal component analysis was performed based on Roger’s distance and calculated using 6734 SNPs

Individuals with multiple favorable haplotypes were identified through ‘stacking’ and the effect on mean trait value observed. As shown in Fig. 5a individuals with six favorable haplotypes identified in the MET had a mean value for scsAUC of 209.4 while those with one significant haplotype at any of the blocks had a mean value of 180.1. There is a greater range of scsAUC for lines with 4 or 5 stay-green haplotypes compared to those with 6. However, this is likely due to only two individuals in the panel possessing all 6 haplotypes. Commercially released cultivars banks, Dash, Oxford, and Maximus CL among others were found to have 5 blocks with stay-green haplotypes while Buloke, Compass, and Fathom contained two. Individuals with a high number of stay-green haplotypes are found to be spread throughout most of the genetic diversity of the population and in all clusters (Fig. 5b). The two lines with all six stay-green haplotypes are found in cluster 3 (modern Australian varieties).

**Fig. 6:**
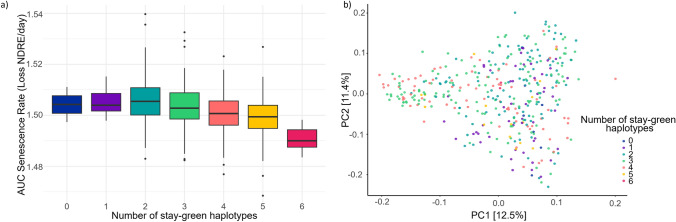
Haplotypes for area under the curve (AUC) of senescence rate identified from best linear unbiased estimators from 22GAT. **a** AUC for individuals grouped by the number of stay-green haplotypes, **b** diversity of genotypes in the barley breeding panel with number of stay-green haplotypes highlighted as per key. Principal component analysis was performed based on Roger’s distance and calculated using 6734 SNPs

Slowing the rate of senescence is the key target for stay-green crops, and as such, identifying haplotypes contributing to rate as well as total greenness is important. In the 22GAT area under the rate curve six blocks with high variance for haplotype effect were identified. As shown in Fig. 6a individuals with 0 stay-green haplotypes had a greater area under the rate curve than those with 6. Individuals with the high number of stay-green haplotypes are found within only clusters one (historic Australian varieties) and two (European varieties) (Fig. 6b).

**Fig. 7:**
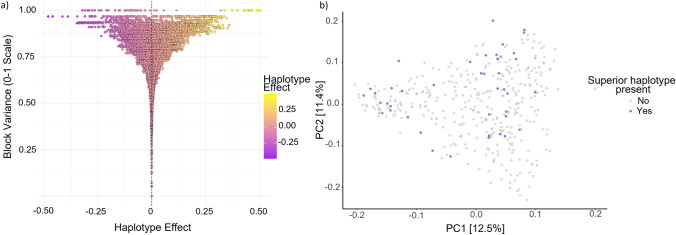
Superior haplotype identification and genetic distribution. **a** The relationship between haplotype variance and effects calculated based on the local genomic estimated breeding value (localGEBV) approach using best linear unbiased estimators (BLUEs) from multi-environment trial analysis of area under senescence curve during grain filling measured across six environments. **b** Distribution of barley genotypes carrying a ‘superior haplotype’ (effect > 0.45) at block b002278

Given the trait-specific nature of variance, the scaled min–max variance was utilized to identify significant blocks in each analysis. Block b002278 had the highest scaled variance in three out of the four analyses it was identified in, as well as large effects as shown in Fig. 7. This block contains haplotypes with the greatest stay-green effect but also several haplotypes with strong negative effects on stay-green. Haplotypes with a total effect of greater than 0.45 were considered superior haplotypes for block b002278 and their distribution among the germplasm panel was explored. These five haplotypes for b002278 were found to be spread throughout the population (Fig. 7b).

**Fig. 8:**
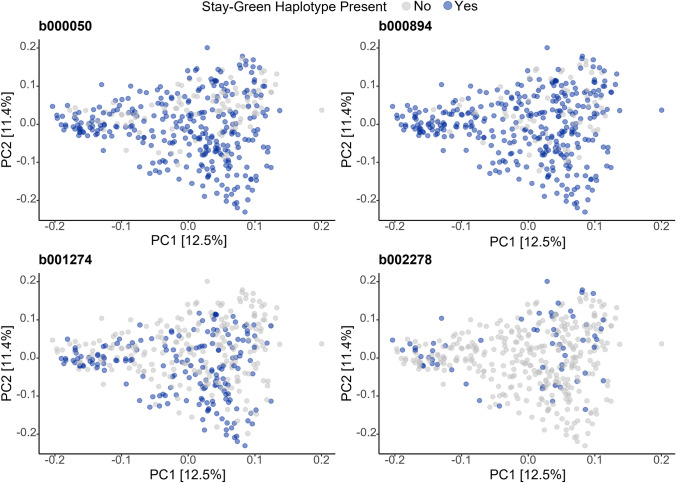
Diversity of barley genotypes with haplotypes associated with stay-green. Diversity of genotypes in the barley breeding panel with stay-green haplotypes at blocks b000050 (Chr 1H), b002278 (Chr 6H), b000894 (Chr 3H), and b001274 (Chr 4H). Principal component analysis was performed based on Roger’s distance and calculated using 6734 SNPs

A stay-green haplotype (any haplotype with effect > 0) for b002278 was found in only 60 individuals in the dataset, however, appear to come from multiple sources (Fig. 8). This includes elite breeding cultivars Banks, Dash, and Lockyer. Both Dash and Lockyer have a superior haplotype for stay-green. Three other blocks were identified to have significant variance across three or more analysis and were selected for further investigation. Block b00050 was identified in multiple analyses, a stay-green haplotype was found in 298 of the genotypes in the panel and across the full range of genetic diversity in the population (Fig. 8). Block b001274 and b000894 were also identified in all three clusters. Individuals with positive haplotypes for all four of these blocks were identified across all clusters; however, the combination of haplotypes is mostly found in cluster 3, additionally there are no individuals in cluster 3 that do not possess positive haplotypes at any of these four blocks.

## Discussion

### Senescence traits have large variation and are strongly influenced by environment

To our knowledge, this is the first study to characterize stay-green in a large barley breeding panel evaluated across multiple environments. Significant variation was observed between environments for area under the senescence curve, which is similar to reports in wheat (Hassan et al. 2021). Among the common commercial cultivars there was large variation in scsAUC, with Maximus CL, Cyclops, and Rosalind having consistently high values (Fig. 3). However, the fastest senescing lines were less consistent with a wide range of lines across environments. This suggests that while stay-green may have a large genetic contribution, environment is more important for accelerating senescence. This could be due to environmental stresses such as heat triggering early onset or faster senescence due to unfavorable conditions for a specific genotype (Hajibarat and Saidi 2022).

For NDRE during grain filling (Fig. 3) no genetic variance was observed for several traits including average rate, area under the rate curve, and area under the curve of the second derivative. It is likely that the limited number of UAV flights during this key development stage limited the capacity to detect variation in NDRE with five of the six sites having only three flights during grain fill. Further investigation into the optimum number and timing of flights to best capture genotypic variation in stay-green would aid in the optimization of phenotyping protocols for deployment into breeding programs. In addition, thermal time used in this study does not accurately align with growth stages across the six environments. This may be the result of photoperiod difference in each environment (Ochagavía et al. 2022). To account for this in future studies photothermal time which includes day length in the calculation should be utilized allowing for improved ability to link growth stages across the range of environments (McMaster et al. 2008).

MET analysis identified significant environmental effects for scsAUC with both crossover and scale type G × E present (Fig. 4). This is to be expected based on previous findings in wheat, which determined that senescence rate is strongly influenced by environmental factors such as water availability (Christopher et al. 2016). Environmental clusters were formed from the common polarity between factors across environments, and analysis of weather data for each environment provided insight into potential reasons for iClass groupings. The PPP iClass experienced significantly higher rainfall than any of the other sites during the month around flowering. The PNP and PNN iClasses do not show any clear patterns as to why the environments would have clustered in this manner, although temperature around flowering was close to optimum for the PNN iClass with both high and low temperatures experienced by the other environments at this time providing a potential cause. Validation of this could be completed through a reaction norm approach partitioning different types of G × E and assigning weather traits to account for environmental variation (Waters et al. 2023).

Given the established relationship between stay-green and drought conditions in other crops, environmental variation for the trait was to be expected. All six environments experienced moderate to high levels of rainfall preventing characterization of stay-green under severe drought conditions. Thus, future research is needed to quantify the value of barley stay-green under a range of drought scenarios. 

### A context-dependent increase in barley yield is influenced by stay-green

The consistently positive correlation between yield and scsAUC is evidence for stay-green as a trait which can increase yield. This was expected based on the previous work on stay-green in barley, however, is the first time this correlation has been shown in a large-scale experiment (Emebiri 2013; Gous et al. 2016; Williams et al. 2022). This positive genetic correlation was maintained across all environments with varying strengths suggesting a degree of context dependency in the relationship (Table 5). The magnitude of phenotypic correlation did not align perfectly with genetic correlation although remained consistently positive. Less senescence during grain fill is linked with increased yield across the range of environments in this study.

Interestingly, 22DND had the largest positive genetic and phenotypic correlation between stay-green and yield. Previous research in wheat has found that under non-water-limited conditions stay-green is negatively correlated with yield (Derkx et al. 2012; Kipp et al. 2014). This contradicts the hypothesis that stay-green may decrease barley yield under high water environments given that 22DND experienced the greatest total rainfall.

Similarly, the 22CGN environment which had the least rainfall over the course of the trial, showed the weakest genetic correlation between yield and scsAUC. While this site received adequate rainfall (not considered a drought environment) it provides an indication there is a weaker, but still positive, relationship between stay-green and yield in drier conditions. The timing of rainfall events may be impacting the relationship here, as crop water requirements vary with growth stage and as such requires further investigation.

### Stay-green in barley is genetically complex but contextually dependent

Previous studies of stay-green in barley found contrasting results regarding its genetic architecture (Emebiri 2013; Gous et al. 2016). In this study, multiple regions were identified for the trait indicating stay-green is under complex genetic control. High variance LD blocks were identified on all chromosomes of barley with five blocks on both chromosomes 3H and 6H. There is overlap between blocks detected by different analyses for different environments, where of the 22 blocks identified, 10 were deemed to have high variance in more than one analysis. One block was stable across all environments, b002278 and was found to have the greatest scaled variance in most environments. This block was detected near the centromeric region of chromosome 6H and may be the same QTL identified as HGSQ by Gous et al. (2016) and 1_0124—Chr 6H by Emebiri (2013). Given the use of different genotyping approaches in each of these studies and lack of exact position it is not possible to conclude if these previous findings are a result of the same underlying gene(s). The identification of five superior haplotypes for block b002278, many of which are already in modern breeding material and cultivars such as Dash and Lockyer, provides an opportunity to explore the impact of incorporating these haplotypes into new varieties.

Three blocks were identified as important in three trials: b000050 (Chr 1H), b000894 (Chr 3H) and b001274 (Chr 4H). Notably block b001274 was identified from the 22GAT complete growth curves for both area under the rate curve and senescence curve providing evidence that the total greenness and loss of chlorophyll can be controlled by overlapping genomic regions. Contrastingly, some blocks were identified to be specific to either area under senescence or rate curve suggesting that different blocks can be associated with different stay-green mechanisms. Investigating this in a multi-environment framework is a future opportunity with increased phenotyping frequency across all environments.

Blocks b000050 and b00894 do not appear to have been previously identified as contributing to barley stay-green, and hence are novel findings for this study. Emebiri (2013) identified a genomic region on chromosome 4H that appears to be in a similar location to block b001274, however, as with b002278, further validation is required.

Given the strong relationship between senescence and flowering it was likely that key flowering genes may be detected in the analysis despite methodology to remove flowering time correlations. The key genes controlling barley flowering time *VRN1* (Chr 5H) and *PPD1* (Chr 2H) were not detected in the analysis (Fernández-Calleja et al. 2021). This provides some assurance that haplotypes identified as contributing to stay-green are not simply altering flowering time.

In keeping with previous research, many blocks were identified to be specific to environments most notably those identified in the PNN iClass rarely overlapped with blocks detected in other analyses. This is likely due to the less than favorable conditions experienced at the sites within this iClass during flowering, indicating strong environmental plasticity for stay-green under these conditions.

### Stay-green in barley is present in multiple genetic backgrounds

For the practical application of these identified blocks, individuals with positive haplotypes for scsAUC were identified. This also provided insight into the potential origin of these haplotypes and by extension any potential of unintentional trait selection during breeding for other traits. The highest concentration of individuals with four or more blocks is found in cluster 3, the modern barley breeding material (Fig. 5b). Hence, despite no current breeding priority for stay-green it can be inferred that due to the correlation between stay-green and traits of breeding significance (i.e., yield) a degree of indirect selection for the trait has occurred.

In contrast, there are no individuals in cluster 3 that possess all 6 negative haplotypes for senescence rate (Fig. 6b). Additionally, the majority of those with 5 senescence rate haplotypes and all with 6 are found in cluster 2 suggesting these haplotypes are primarily present in European barley genotypes. This provides an opportunity to further delay the onset of senescence in modern Australian breeding lines through future crosses. Further investigation into the reason for the haplotypes absence in modern varieties needs to be conducted before a decision could be made regarding the merits of reintroducing high numbers of haplotypes for increased senescence rate into modern Australian barley breeding lines.

### In silico haplotype stacking reveals potential to improve senescence traits through breeding

The simulated stacking of favorable haplotypes showed a clear linear relationship for area under senescence curve (Fig. 5a), showing the benefit of including multiple desirable haplotypes when aiming to develop stay-green barley varieties. While a linear relationship was also evident for area under senescence rate curve haplotypes (Fig. 6a), the differences associated with stacking multiple haplotypes were smaller and the true relationship was less obvious. Notably, this analysis was conducted using data from only one well-watered environment, thus limits the power to draw conclusions. Richer UAV datasets across multiple environments with different rainfall patterns are required to explore the haplotype combinations more thoroughly.

The molecular nature of stay-green is known to be multifaceted with several traits intersecting for this phenotype. As such, understanding the biological mechanisms associated with each haplotype would enable prioritization of complementary stay-green approaches to understand any potential tradeoffs and potentially reduce haplotype number required for significant trait improvement.

Breeding six haplotypes into a population using marker-assisted selection would be costly and time consuming. Currently, breeders are leaning toward a whole genome approach to barley breeding with emphasis on genomic selection. Since stay-green has been identified to be highly complex and environmentally dependent trait, this approach would likely be the most efficient to facilitate selection for the trait in routine breeding operations. However, haplotype-based selection at the 6H region may be beneficial given its large effect and environmental stability. Further research is required to determine the mechanisms associated with the role of region 6H in stay-green.

Combining the stay-green haplotypes in silico highlighted the potential for breeders to fine tune senescence profiles to suit a range of environments. Stay-green is an exceptional candidate for selection in barley breeding programs given its promise as a key drought adaptation trait with the potential to improve yields under a range of environments, which is an essential consideration for breeding in future climates.

### Supplementary Information

Below is the link to the electronic supplementary material.

Supplementary file1 (DOCX 4369 KB)

## Data Availability

The data that support the findings of this study are available from InterGrain Pty Ltd, but restrictions apply to the availability of these data, which were used under license for the current study, and so are not publicly available. Data are, however, available from the authors upon reasonable request and with permission of InterGrain Pty Ltd.

## References

[CR1] Akaike H (1974). A new look at the statistical model identification. IEEE Trans Autom Control.

[CR2] Amadeu RR, Garcia AAF, Munoz PR, Ferrão LFV (2023). AGHmatrix: genetic relationship matrices in R. Bioinformatics.

[CR3] Anderegg J, Yu K, Aasen H, Walter A, Liebisch F, Hund A (2020). Spectral vegetation indices to track senescence dynamics in diverse wheat germplasm. Front Plant Sci.

[CR4] Badea A, Wijekoon C (2021). Benefits of barley grain in animal and human diets. Cereal Grains.

[CR5] Borrell AK, Hammer GL, Henzell RG (2000). Does maintaining green leaf area in sorghum improve yield under drought? II. dry matter production and yield. Crop Sci.

[CR6] Borrell AK, Mullet JE, George-Jaeggli B, van Oosterom EJ, Hammer GL, Klein PE, Jordan DR (2014). Drought adaptation of stay-green sorghum is associated with canopy development, leaf anatomy, root growth, and water uptake. J Exp Biol.

[CR43] Balazadeh S (2014). Stay-green not always stays green. Mol Plant.

[CR7] Butler D, Cullis BR, Gilmour A, Gogel B (2009) ASReml-R reference manual. The State of Queensland, Department of primary industries and fisheries, Brisbane

[CR8] Christopher JT, Christopher MJ, Borrell AK, Fletcher S, Chenu K (2016). Stay-green traits to improve wheat adaptation in well-watered and water-limited environments. J Exp Bot.

[CR9] S Das SR Massey-Reed J Mahuika J Watson C Cordova L Otto Y Zhao S Chapman B George-Jaeggli D Jordan GL Hammer AB Potgieter 2022 A high-throughput phenotyping pipeline for rapid evaluation of morphological and physiological crop traits across large fields. IEEE International Geoscience and Remote Sensing Symposium 2022, pp 7783–7786 10.1109/IGARSS46834.2022.9884530

[CR10] Derkx AP, Orford S, Griffiths S, Foulkes MJ, Hawkesford MJ (2012). Identification of differentially senescing mutants of wheat and impacts on yield, biomass and nitrogen partitioning. J Integr Plant Biol.

[CR11] Emebiri LC (2013). QTL dissection of the loss of green colour during post-anthesis grain maturation in two-rowed barley. Theor Appl Genet.

[CR12] Feller U, Fischer A (2011). Nitrogen metabolism in senescing leaves. Crit Rev Plant Sci.

[CR13] Fernández-Calleja M, Casas AM, Igartua E (2021). Major flowering time genes of barley: allelic diversity, effects, and comparison with wheat. Theor Appl Genet.

[CR14] Gous PW, Hickey L, Christopher JT, Franckowiak J, Fox GP (2016). Discovery of QTL for stay-green and heat-stress in barley (*Hordeum vulgare*) grown under simulated abiotic stress conditions. Euphytica.

[CR15] Hajibarat Z, Saidi A (2022). Senescence-associated proteins and nitrogen remobilization in grain filling under drought stress condition. J Genet Eng Biotechnol.

[CR16] Hassan MA, Yang M, Rasheed A, Tian X, Reynolds M, Xia X, Xiao Y, He Z (2021). Quantifying senescence in bread wheat using multispectral imaging from an unmanned aerial vehicle and QTL mapping. Plant Physiol.

[CR17] Jeffrey SJ, Carter JO, Moodie KB, Beswick AR (2001). Using spatial interpolation to construct a comprehensive archive of Australian climate data. Environ Model Softw.

[CR18] Kamal NM, Alnor Gorafi YS, Abdelrahman M, Abdellatef E, Tsujimoto H (2019). Stay-green trait: a prospective approach for yield potential, and drought and heat stress adaptation in globally important cereals. Int J Mol Sci.

[CR19] Keeble-Gagnere G, Pasam R, Forrest KL, Wong D, Robinson H, Godoy J, Rattey A, Moody D, Mullan D, Walmsley T, Daetwyler HD, Tibbits J, Hayden MJ (2021). Novel design of imputation-enabled SNP arrays for breeding and research applications supporting multi-species hybridization. Front Plant Sci.

[CR20] Kipp S, Mistele B, Schmidhalter U (2014). Identification of stay-green and early senescence phenotypes in high-yielding winter wheat, and their relationship to grain yield and grain protein concentration using high-throughput phenotyping techniques. Funct Plant Biol.

[CR21] Liang X, Liu Y, Chen J, Adams C (2017). Late-season photosynthetic rate and senescence were associated with grain yield in winter wheat of diverse origins. J Agron Crop Sci.

[CR22] Liedtke JD, Hunt CH, George-Jaeggli B, Laws K, Watson J, Potgieter AB, Cruickshank A, Jordan DR (2020). High-throughput phenotyping of dynamic canopy traits associated with stay-green in grain sorghum. Plant Phenomics.

[CR23] Liu J, Zhu Y, Tao X, Chen X, Li X (2022). Rapid prediction of winter wheat yield and nitrogen use efficiency using consumer-grade unmanned aerial vehicles multispectral imagery. Front Plant Sci.

[CR24] Lopes MS, Reynolds MP (2012). Stay-green in spring wheat can be determined by spectral reflectance measurements (normalized difference vegetation index) independently from phenology. J Exp Bot.

[CR25] McMaster GS, White JW, Hunt LA, Jamieson PD, Dhillon SS, Ortiz-Monasterio JI (2008). Simulating the influence of vernalization, photoperiod and optimum temperature on wheat developmental rates. Ann Bot.

[CR26] Meuwissen TH, Hayes BJ, Goddard ME (2001). Prediction of total genetic value using genome-wide dense marker maps. Genetics.

[CR27] Ochagavía H, Kiss T, Karsai I, Casas AM, Igartua E (2022). Responses of barley to high ambient temperature are modulated by vernalization. Front Plant Sci.

[CR28] Panday US, Pratihast AK, Aryal J, Kayastha RB (2020). A review on drone-based data solutions for cereal crops. Drones.

[CR29] Perez-Valencia DM, Rodriguez-Alvarez MX, Boer MP, Kronenberg L, Hund A, Cabrera-Bosquet L, Millet EJ, Eeuwijk FAV (2022). A two-stage approach for the spatio-temporal analysis of high-throughput phenotyping data. Sci Rep.

[CR30] R Development Core Team (2023) R: A language and environment for statistical computing. R Foundation for Statistical Computing, Vienna, Austria

[CR31] Rogers J (1972). Meaures of gentic similarity and genetic distance. Studies Genet VII.

[CR32] Selection tools developers (2023) SelectionTools: SelectionTools.

[CR33] Shin D, Lee S, Kim TH, Lee JH, Park J, Lee J, Lee JY, Cho LH, Choi JY, Lee W, Park JH, Lee DW, Ito H, Kim DH, Tanaka A, Cho JH, Song YC, Hwang D, Purugganan MD, Jeon JS, An G, Nam HG (2020). Natural variations at the stay-green gene promoter control lifespan and yield in rice cultivars. Nat Commun.

[CR34] Shirdelmoghanloo H, Chen K, Paynter BH, Angessa TT, Westcott S, Khan HA, Hill CB, Li C (2022). Grain-filling rate improves physical grain quality in barley under heat stress conditions during the grain-filling period. Front Plant Sci.

[CR35] Smith A, Cullis B, Thompson R (2001). Analyzing variety by environment data using multiplicative mixed models and adjustments for spatial field trend. Biometrics.

[CR36] Smith AB, Ganesalingam A, Kuchel H, Cullis BR (2015). Factor analytic mixed models for the provision of grower information from national crop variety testing programs. Theor Appl Genet.

[CR37] Voss-Fels KP, Stahl A, Wittkop B, Lichthardt C, Nagler S, Rose T, Chen TW, Zetzsche H, Seddig S, Majid Baig M, Ballvora A, Frisch M, Ross E, Hayes BJ, Hayden MJ, Ordon F, Leon J, Kage H, Friedt W, Stutzel H, Snowdon RJ (2019). Breeding improves wheat productivity under contrasting agrochemical input levels. Nat Plants.

[CR38] Waters DL, van der Werf JHJ, Robinson H, Hickey LT, Clark SA (2023). Partitioning the forms of genotype-by-environment interaction in the reaction norm analysis of stability. Theor Appl Genet.

[CR39] Williams JL, Sherman JD, Lamb P, Cook J, Lachowiec JA, Bourgault M (2022). Relationships between roots, the stay-green phenotype, and agronomic performance in barley and wheat grown in semi-arid conditions. The Plant Phenome J.

[CR40] Yang H, Huang T, Ding M, Lu D, Lu W (2017). High Temperature during grain filling impacts on leaf senescence in waxy maize. Agron J.

[CR41] Zadoks JC, Chang TT, Konzak CF (1974). A decimal code for the growth stages of cereals. Weed Res.

[CR42] Zhang W, Peng K, Cui F, Wang D, Zhao J, Zhang Y, Yu N, Wang Y, Zeng D, Wang Y, Cheng Z, Zhang K (2021). Cytokinin oxidase/dehydrogenase OsCKX11 coordinates source and sink relationship in rice by simultaneous regulation of leaf senescence and grain number. Plant Biotechnol J.

