# Supplementary material for: Characterizing stay-green in barley across diverse environments: unveiling novel haplotypes
Source: Theor Appl Genet. 2024 May 6;137(6):120. doi: 10.1007/s00122-024-04612-1 (PMC11074220; doi:10.1007/s00122-024-04612-1)
Supplement: Supplementary file 1 — Supplementary file1 (DOCX 4369 KB) [file 122_2024_4612_MOESM1_ESM.docx]

**
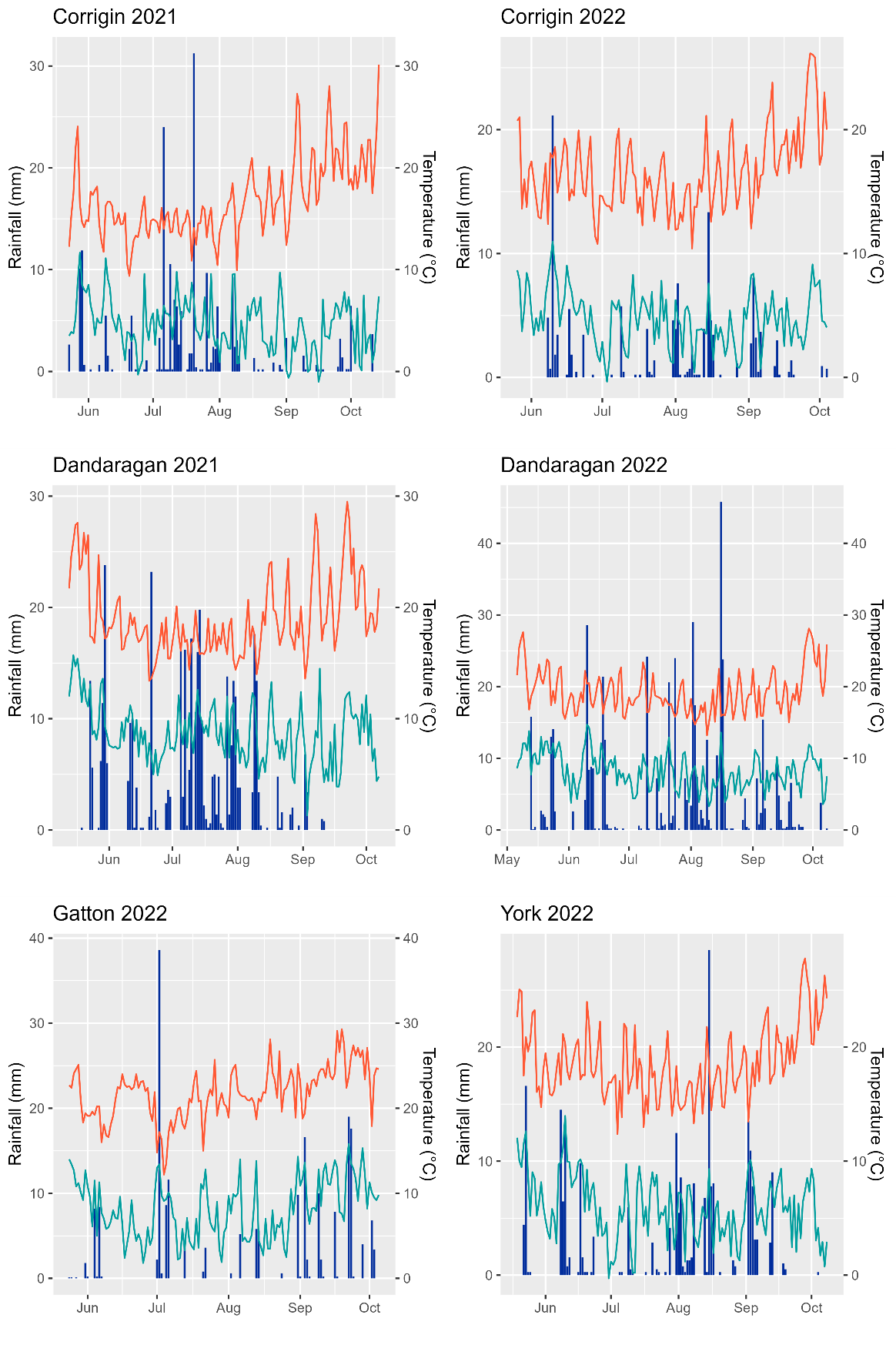
Supplementary Figures**

**Fig. S1: Weather Data for all sites during trial season from sowing to final UAV flight.** Red line indicates maximum daily temperature, blue line indicates minimum daily temperature and blue bars represent daily rainfall.

**
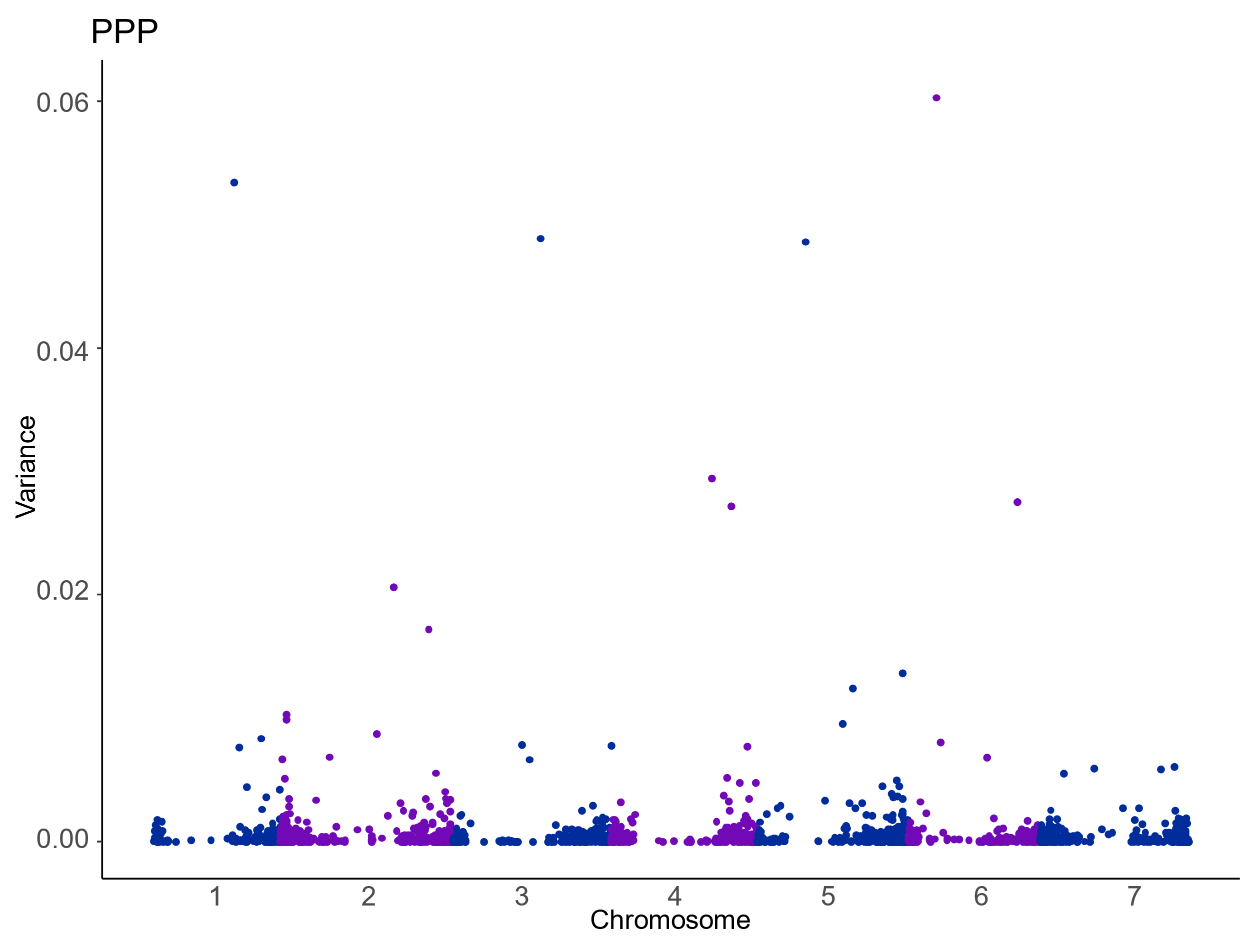

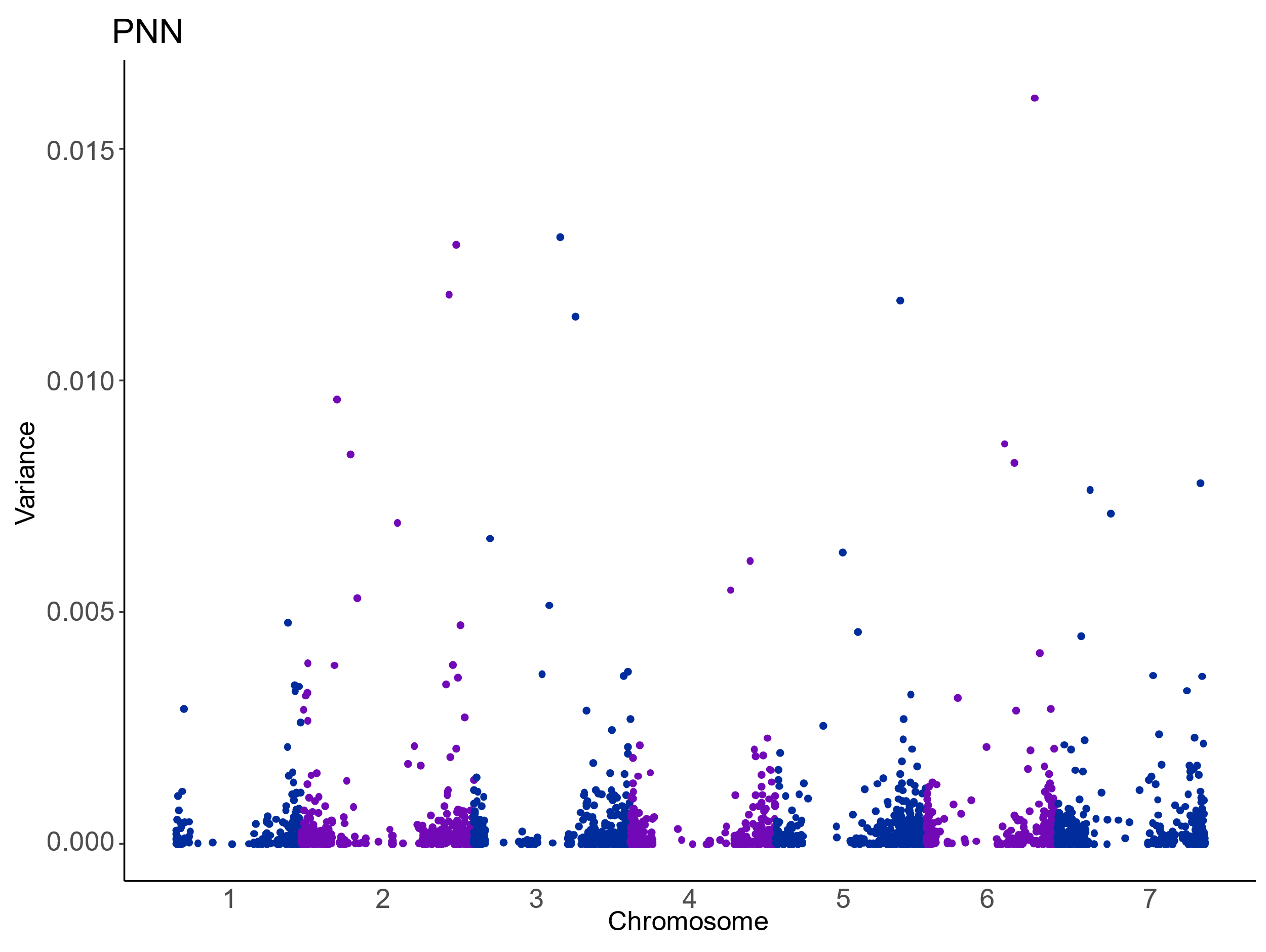

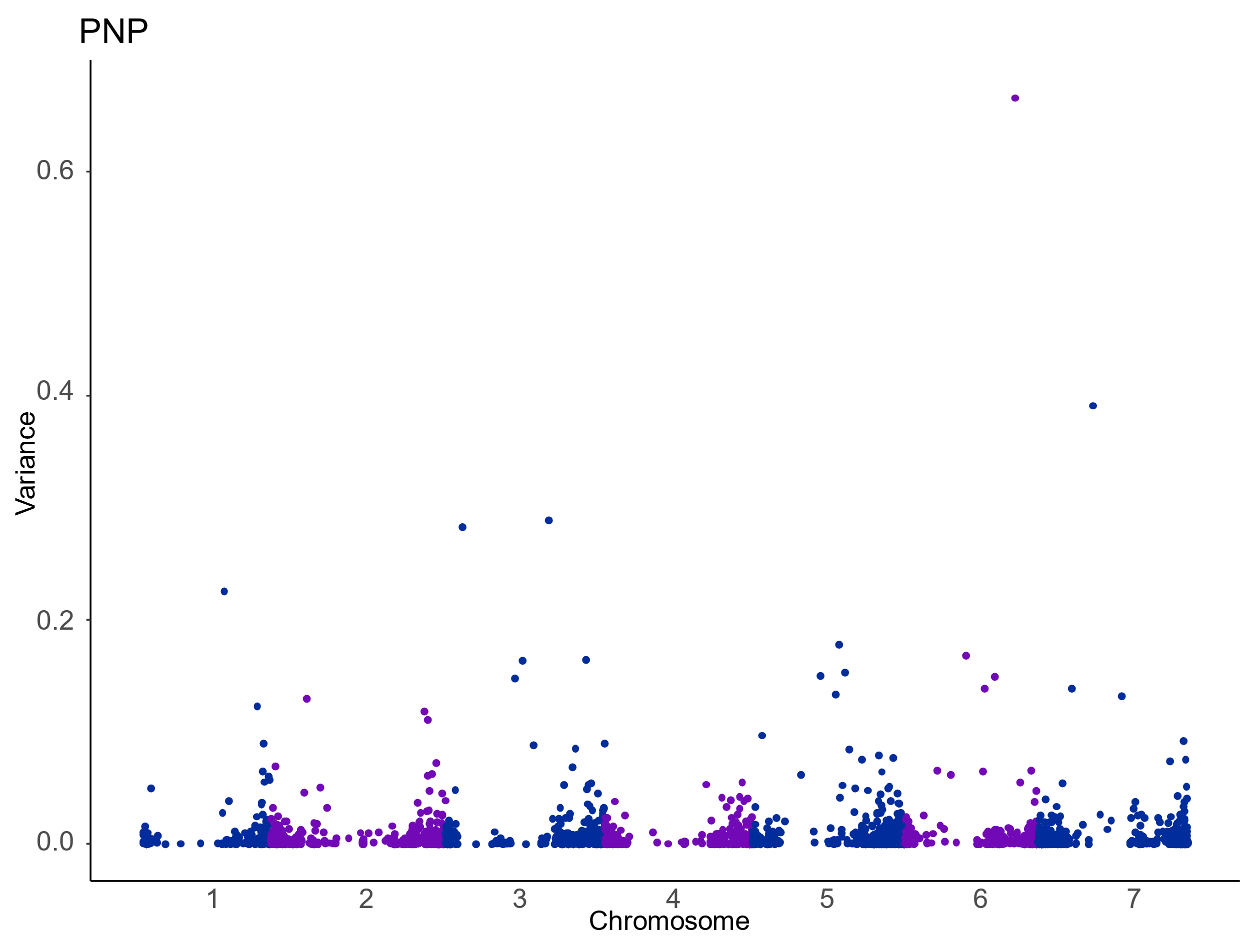

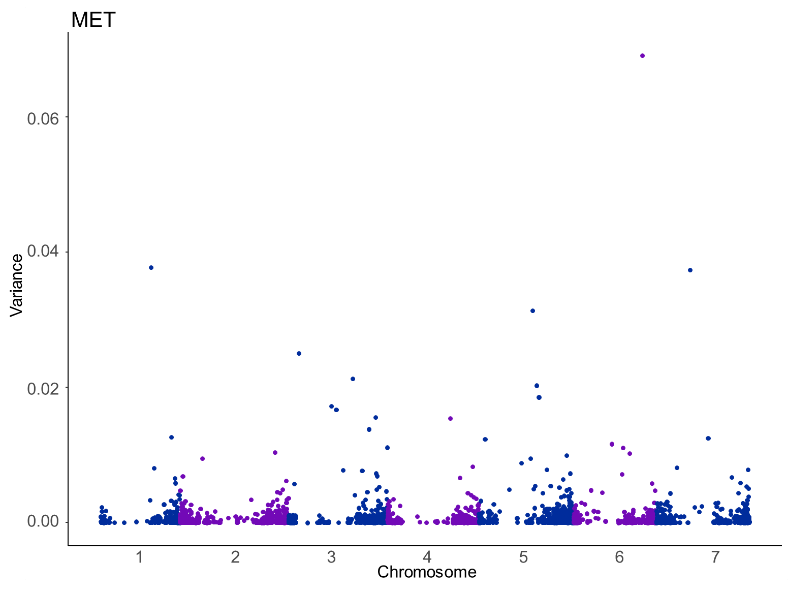
**

**Fig. S2: Manhattan plots of variance per block for area under senescence curve.** Variance per block calculated utilising BLUEs from the MET and BLUEs from each iClass (PNN, PNP, PPP).


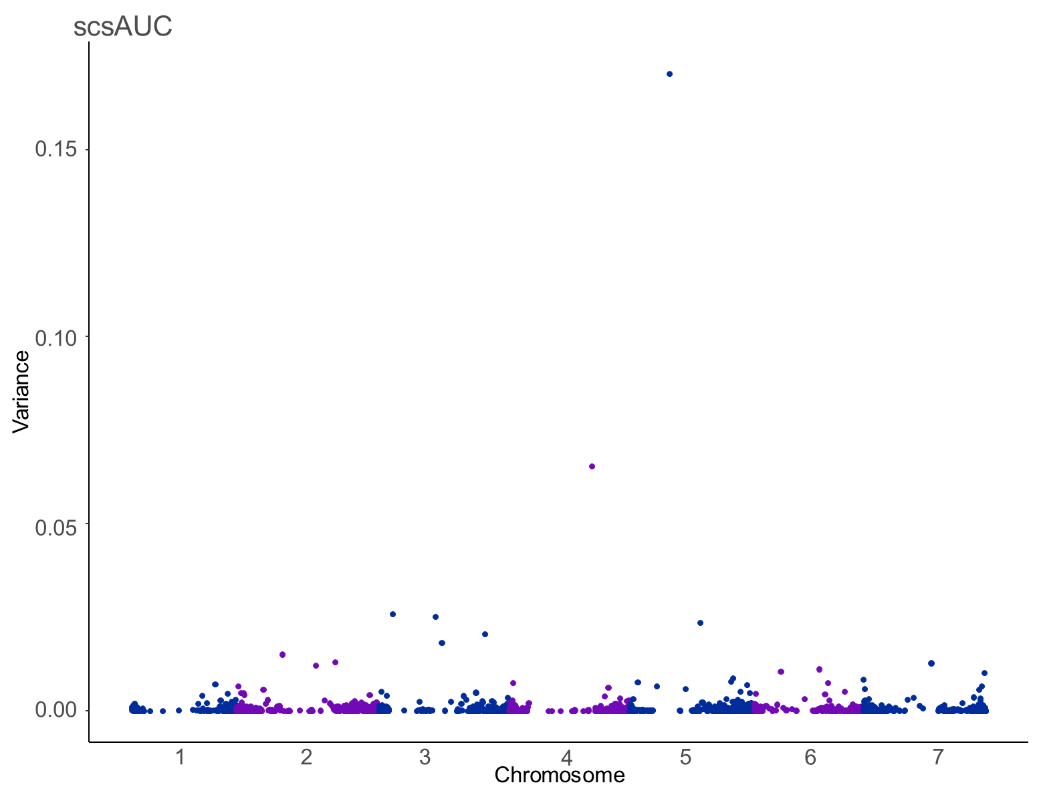

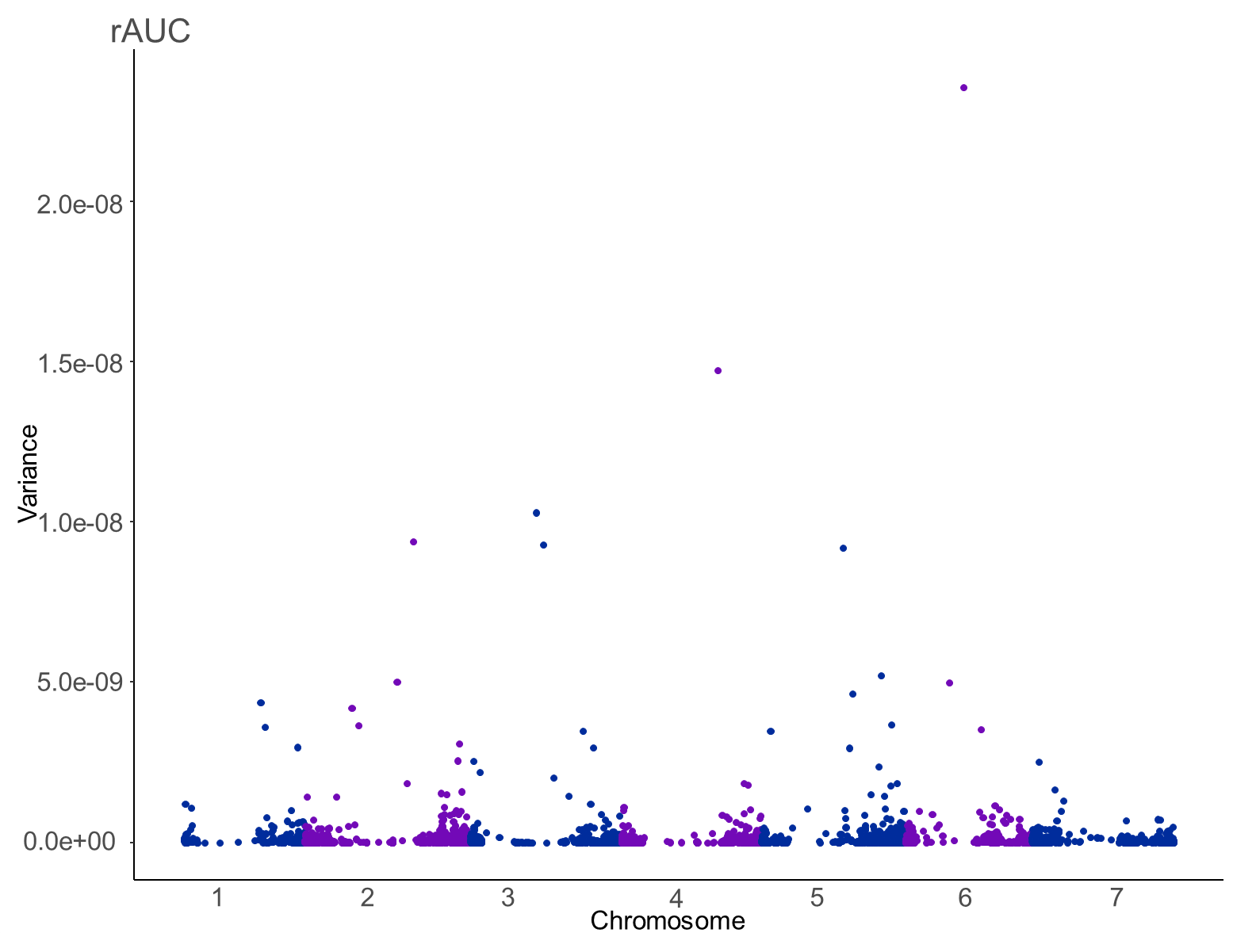


**Fig. S3: Manhattan plots of variance per block for Gatton senescence profile.** Variance per block calculated utilising BLUEs from 22GAT spatiotemporal analysis for area under senescence curve (scsAUC) and area under rate curve (rAUC).
